# Five-Year Predictors of Insulin Initiation in People with Type 2 Diabetes under Real-Life Conditions

**DOI:** 10.1155/2018/7153087

**Published:** 2018-09-19

**Authors:** Sandro Gentile, Felice Strollo, Francesca Viazzi, Giuseppina Russo, Pamela Piscitelli, Antonio Ceriello, Carlo Giorda, Piero Guida, Paola Fioretto, Roberto Pontremoli, Salvatore De Cosmo, the AMD-Annals Study Group

**Affiliations:** ^1^Department of Clinical and Experimental Medicine, University of Campania “Luigi Vanvitelli”, Naples, Italy; ^2^Diabetes Center, San Raffaele Termini, Rome, Italy; ^3^Department of Internal Medicine, University of Genoa and Policlinico San Martino, Genova, Italy; ^4^Department of Clinical and Experimental Medicine, University of Messina, Messina, Italy; ^5^IRCSS Casa Sollievo della Sofferenza-Unit of Internal Medicine, Scientific Institute, San Giovanni Rotondo, Italy; ^6^Institut d'Investigacions Biomédiques August Pi i Sunyer (IDIBAPS), Centro de Investigación Biomedica en Red de Diabetes y Enfermedades Metabólicas Asociadas (CIBERDEM), Barcelona, Spain; ^7^Department of Cardiovascular and Metabolic Diseases, IRCCS Multimedica, Milan, Italy; ^8^Diabetes and Metabolism Unit ASL Turin 5, Chieri, Italy; ^9^Statistical Consultant, Associazione Medici Diabetologi (AMD), Rome, Italy; ^10^Department of Medicine, University of Padua, Padua, Italy; ^11^Associazione Medici Diabetologi (AMD), Rome, Italy

## Abstract

We performed a real-life analysis of clinical and laboratory parameters, in orally treated T2DM patients aiming at identifying predictors of insulin treatment initiation. Overall, 366955 patients (55.8% males, age 65 ± 11 years, diabetes duration 7 ± 8 years) were followed up between 2004 and 2011. Each patient was analyzed step-by-step until either eventually starting insulin treatment or getting to the end of the follow-up period. Patients switching to insulin showed a worse global risk profile, longer disease duration (10 ± 9 years vs. 6 ± 7 years, respectively; *p* < 0.001), higher HbA1c (8.0 ± 1.6% vs. 7.2 ± 1.5%, respectively; *p* < 0.001), higher triglycerides, a greater prevalence of arterial hypertension, antihypertensive, lipid-lowering and aspirin treatment, a higher rate of nonproliferative/proliferative retinopathy, and a nearly 4 times lower prevalence of the “diet alone.” They also showed a higher prevalence of subjects with eGFR < 60 ml/min/1.73 m^2^ (24.0% vs. 16.2%, respectively; *p* < 0.001). Multivariate analysis identified diabetes duration, HbA1c, triglyceride and low HDL-C values, presence of retinopathy or renal dysfunction, and sulphonylurea utilization (the risk being approximately 3 times greater in the latter case) as independent predictors of insulin treatment initiation. LDL-C, lipid-lowering treatment, and overweight/obese seem to be protective. Results of tree analysis showed that patients on sulphonylurea, with high HbA1c, eGFR below 50 ml/min/1.73 m^2^, and at least 5-year disease duration, are at very high risk to start insulin treatment. We have to stick to this real-life picture, of course, until enough data are collected on patients treated with innovative medications which are expected to improve beta cell survival and further delay treatment-related insulin requirement.

## 1. Introduction

Type 2 diabetes mellitus (T2DM) is a chronic disease, characterized by insulin resistance and gradual loss of beta cell function, though the relative weight of these mechanisms may change as disease advances [1, 2]. Due to that the answer to “*will I need insulin?*,” one of the most common questions posed to the doctor at the time of diagnosis, varies widely. In addition, patients with T2DM are characterized by different rates of disease progression; this often prevents them from starting insulin treatment for many years if at all [1–4].

The United Kingdom Prospective Diabetes Study (UKPDS) showed that beta cell functional reserve had already halved by the time of diagnosis and continued to decline over the following 6-year observation period on antihyperglycemic therapy [5]. Nevertheless, the rate of beta cell failure is unpredictable in the individual patient; thus, some people with T2DM will never require insulin to treat their blood glucose to target the desired glucose level. In fact, lifestyle, clinical, and metabolic factors and ongoing therapies may have a bearing on beta cell function.

The ADA guidelines [6] suggest early treatment of hyperglycemia in T2DM addressed to individualized targets. It might be realized either by starting from lifestyle measures and then adding one or more drugs stepwise at 3- to 6-month intervals until the adoption of insulin therapy or by prescribing early insulin treatment in specific conditions. The usual sequence of add-on treatments prior to insulin initiation is affected by many factors and is variable in speed from person to person. With the start of insulin therapy, some changes occur in perceived quality of life, the risk of hypoglycemia, and/or weight gain increases and a more stringent self-monitoring blood glucose is required, with major effects on costs, care management, and lifestyle. Therefore, early identification of clinical predictors of insulin treatment initiation could provide clues on best possible strategies aimed at improving the long-term management of T2DM.

Other studies already tried to engage with such an intriguing task, but limited case series and/or the presence of clinical factors eventually limiting the use of blood glucose-lowering drugs other than insulin did not allow drawing firm conclusions on this issue [7–9]. Renal function, as expressed by glomerular filtration rate (GFR), progressively decreases with time and is expected to be one of the most relevant factors leading to insulin therapy [10].

We therefore took advantage of the large AMD Annals initiative database [11] to perform a real-life analysis of clinical and laboratory parameters, complications, and patterns of glucose-lowering treatment in noninsulin-requiring T2DM patients with the aim of correlating to age and baseline renal function, the pattern of insulin treatment initiation eventually occurring during follow-up.

## 2. Materials and Methods

### 2.1. Study Design, Study Patients, and Data Sources

In the present report, we analyze a large series of patients with T2DM followed up by 301 diabetes centers (DC) homogeneously distributed throughout the country among those affiliated to the Italian Association of Clinical Diabetologists (AMD) initiative with the aim of investigating possible predictors of future insulin treatment initiation according to age and kidney function. From the databases pertaining to those 301 DCs, 510182 people with T2DM were selected at their first estimated GFR (eGFR) evaluation, but immediately after, those already on insulin (*n* = 141832) or less than 18 years old (*n* = 39) or assisted by DCs with <100 patients were excluded to minimize selection bias. So, in the end, 366955 noninsulin-treated T2DM patients followed up by 267 DCs entered the study (flow chart, Supplementary Figure 1). The analysis was performed using the data set of electronic medical records collected between 2004 and 2011.

### 2.2. Data Collection

As already reported, the analysis of the database is an ongoing Italian AMD Annals initiative aimed at identifying a set of indicators to be used and consistently fine-tuned in the context of continuous quality improvement. Participating DCs adopted the same computer program for the everyday management of outpatients, while a dedicated software package allowed us to extract and analyze needed information from the database (AMD data file). The latter in fact collected information exclusively from patients who preliminarily signed their informed consent to the anonymous utilization of their own data for clinical evaluations targeted at diabetes community health and quality of life improvement.

The results were internally approved by the AMD Annals scientific committee. The diagnosis of type 2 diabetes was made/confirmed at each participating DC according to criteria defined by the ADA Standards of Medical Care in Diabetes 2014 [6]. The International Classification of Diseases, Clinical Modification (ICD-9-CM, V82.9 2014) was used to define T2DM diagnosis and comorbidities and/or complications [12].

The AMD Annals Initiative included measuring and regularly recording HbA1c, blood pressure (BP), low-density lipoprotein cholesterol (LDL-c), total and high-density lipoprotein cholesterol (TC and HDL-c, respectively), triglycerides (TG), and serum uric acid (SUA) by high standard autoanalyzers in public laboratories successfully participating in nationwide quality control programs. The use of specific classes of drugs (metformin, other antihyperglycemic agents (AHA), statins, and antihypertensive drugs) was also evaluated. Kidney function was assessed by serum creatinine and urinary albumin excretion measurements. eGFR was calculated for each patient using a standardized serum creatinine assay and the CKD-EPI equation [13].

To be included in the study, each patient had to provide at least one measurement of serum creatinine, with concordant eGFR values, in the 3 months prior to study entry. Increased urinary albumin excretion (UAE) was diagnosed as (i) microalbuminuria if urinary albumin concentration was >30 and ≤300 mg/l or if UAE rate was >20 and ≤200 *μ*g/min or if urinary albumin-to-creatinine ratio (ACR) was >2.5 mg/mmol in men and >3.5 mg/mmol in women and ≤30 mg/mmol in both genders and (ii) macroalbuminuria if urinary albumin concentration was >300 mg/l or if UAE rate was >200 *μ*g/min or if ACR was >30 mg/mmol in both genders. Albuminuria indicated patients with either micro- or macroalbuminuria.

Each enrolled patient entering the study was analyzed step-by-step until either eventually starting insulin treatment or getting to the end of the follow-up period. Six months was the input cut-off in the study, so that the follow-up period ranged from 6 to 96 months, the latter being the case for patients joining already in 2004 and keeping there until 2011 (8 years). As this survey was realized under real-life conditions, a variable amount of missing data occurred for each patient at any steps of the follow-up; the sample size contributing to every follow-up step is given systematically in all result tables.

### 2.3. Statistical Analysis

Data are shown as mean values ± standard deviation. Categorical variables are given as frequencies and percentages. Comparisons were performed by mixed models with clinics fitted as random effect to take into account possible differences across centers. Time to insulin treatment analysis was performed by means of the Cox proportional hazard model with stratification by DCs. Several multivariate models, including an incremental number of variables over subgroups of patients with all data available, were performed to take into account the different completeness of patients' features. Hazard ratios (HRs) were provided for each baseline characteristic. In details, model 1 was based on a limited number of factors over most of the patients. Models 2 to 4 introduced other risk factors, but the sample size decreased. The full model 4 had 44.611 patients in comparison to model 1 that included 323.769 patients. Variables were categorized by clinical cut-point values. *p* values < 0.05 were taken as statistically significant. The cumulative incidence curves for insulin treatment were based on Kaplan–Meier analysis. To define a hierarchical event risk tree, the Cox proportional hazard model was utilized for insulin therapy initiation to recursively split the data into different subgroups by selecting variables characterized by the least *p* values. Continuous variables were analyzed for all values ranging 5th to 95th percentile by selecting the best cut-point with the lowest *p* value. The tree-building process was stopped after three iterations yielding eight groups. All analyses were performed using STATA software, version 14 (Stata-Corp LP, College Station, TX).

## 3. Results

Three hundred and sixty-five thousand nine hundred and fifty-five individuals with T2DM from two hundred and sixty-seven DCs were evaluated. Overall, 54220 patients started insulin therapy at follow-up. Cumulative incidence of insulin treatment initiation was as follows: 1 year 2.5%, 2 years 6.1%, 3 years 10.3%, 4 years 14.5%, 5 years 19.6%, 6 years 24.7%, 7 years 30.1%, and 8 years 36.3%.

The median follow-up with interquartile range was 42 months (21–67), reflecting the fact that half of the patients were followed up for at least 42 months, 25% for 21 months, and 75% for 67 months, respectively.

### 3.1. Baseline Clinical Characteristics of the Whole Population and Stratified by Insulin Treatment during Follow-up

Clinical features of the whole study sample are reported in Table 1. Overall, entry age was 65 ± 11 years, diabetes duration was 7 ± 8 years, BMI was 30 ± 5 kg/m^2^, and 55.8% patients were males. Glycemic control, lipid parameters, and BP levels were fairly good, with mean HbA1c, LDL-C, and systolic/diastolic BP values of 7.3 ± 1.6%, 118 ± 36 mg/dl, and 140 ± 19/81 ± 10 mmHg, respectively. Mean eGFR was 78 ± 19 ml/min/1.73 m^2^. 5.3% and 1.2% people had nonproliferative and proliferative retinopathy, respectively; 38.3% were on lipid-lowering agents (34.7% on statins and 2.3% on fibrates), 58.4% on antihypertensive treatment, and 16.6% on aspirin. As for glycemic control, 11.5% patients were on diet alone (*n* = 42056), 27.2% on metformin (*n* = 99718), 43.5% on metformin + sulphonylurea (*n* = 159664), 17% on sulphonylurea alone (*n* = 62276), and 0.9% on pioglitazone (*n* = 3241). Due the time course of the study—starting at the very beginning of incretin utilization and closing before SGLT2 inhibitor availability—only a minority of patients were taking incretins and no one was on SGLT2 inhibitors.

Table 1 also summarizes baseline clinical characteristics of the 54220 patients (15% of the studied population) who were treated with insulin by the end of follow-up versus their noninsulin-treated counterpart. Patients in the former group showed a worse global risk profile and, despite a higher prevalence of subjects with eGFR < 60 ml/min/1.73 m^2^ (24.0% vs. 16.2%, respectively; *p* < 0.001), were more likely to receive a prescription for sulphonylurea, either alone (19.4% vs. 16.5%, respectively; *p* < 0.001) or in association with metformin (65.7% vs. 39.7%, respectively; *p* < 0.001). As clearly shown in the table, the subgroup of patients treated with insulin also showed longer disease duration (10 ± 9 years vs. 6 ± 7 years, respectively; *p* < 0.001), higher HbA1c (8.0 ± 1.6% vs. 7.2 ± 1.5%, respectively; *p* < 0.001), higher triglycerides (both in terms of mean values and of percentage of subjects with values > 150 mg/dl), a greater prevalence of arterial hypertension, and a higher rate of nonproliferative/proliferative retinopathy. The insulin-treated group also displayed a significantly higher prevalence of concomitant antihypertensive, lipid-lowering and aspirin treatment, and a nearly 4 times lower prevalence of the “diet alone” approach.

Clinical baseline characteristics are reported in Supplementary Table 1. Patients were stratified according to follow-up duration until insulin treatment started. A clear relationship between overall cardiovascular and metabolic risk profile and time to insulin treatment is evident; a worse baseline profile is associated with a shorter follow-up before switching to insulin. People on pioglitazone or metformin alone, as well as those with elevated uric acid levels or antihypertensive treatment, were unlikely to receive insulin treatment at follow-up.

### 3.2. Multivariate Cox Regression Analysis for Insulin Treatment during Follow-up

Table 2 displays the results of the multivariate Cox regression analysis for insulin treatment during follow-up according to four different models ordered by increasing data completeness. A relatively small sample (*n* = 44611) eventually contributed to the complete model, which identified all variables significantly and independently associated to insulin treatment initiation, including known diabetes duration (rather than age per se), HbA1c, triglyceride and low HDL-C values, and the presence of retinopathy or renal dysfunction (reduced eGFR or the presence of micro-/macroalbuminuria). On the other hand, LDL-C and lipid-lowering treatment were associated with a greater chance of not initiating insulin treatment over the study period. Overweight and obese status also seem to decrease the risk. Moreover, as for hypoglycemic agent utilization, when diet-treated subjects are taken as the reference group, the risk of switching to insulin was 2.8 to 3.6 times greater for people on sulphonylurea (alone or combined with metformin, respectively).

### 3.3. Use of Statin Seems to Protect From Insulin Treatment Initiation

The incidence of insulin treatment initiation during follow-up, on the basis of the type of initial therapeutic (glucose-lowering) treatment, is described in Supplementary Figure 2. As already said, the risk was much higher in case of sulphonylurea, either alone or in association with metformin, while it remained rather low and almost stable over time in patients on diet or glitazones or metformin alone.

Baseline mean ± SD values or absolute rates (percentage) of all the investigated parameters are also reported on the basis of hypoglycemic treatment. From this point of view, many patients with eGFR < 60 ml/min/1.73 m^2^ were on sulphonylurea alone (n. 18983; 30.5%) or associated with metformin (n. 26465; 16.6%), while only 446 of 1806 (13.8) were under pioglitazone (Supplementary Table 2).

The cumulative incidence of insulin treatment initiation during follow-up was greater in patients with eGFR < 60 ml/min/1.73 m^2^ at baseline in comparison to those with 60–90 or >90 which had a similar incidence (Supplementary Figure 3).

**Figure 1:**
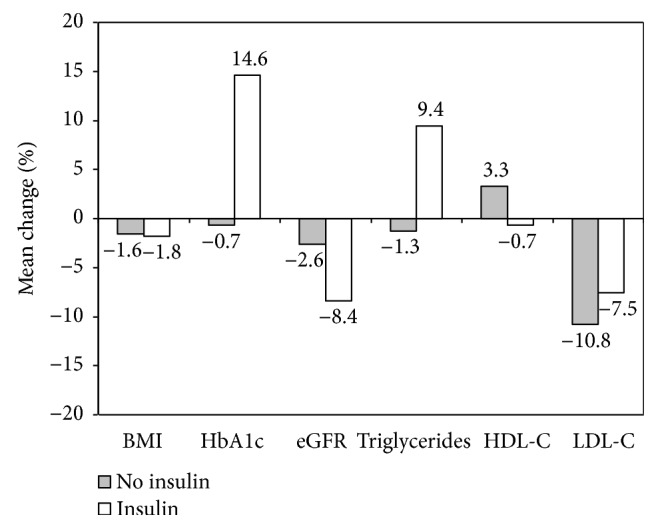
Change (%) from baseline by insulin treatment of main significant parameters identified by multivariate analysis. All differences were statistically significant (*p* < 0.001). Patients with less than one-year follow-up duration were excluded. Numbers of paired observations at baseline and final visits were: BMI 193238, HbA1c 227368, eGFR 183858, Triglycerides 171670, HDL-C 164966, LDL-C 160112, respectively.

In Figure 1, the mean changes from baseline (%) (±SD) are shown for all parameters found to be significant by multivariate analysis. All of them were significantly worse at the end of follow-up in insulin-treated patients (*p* < 0.001).

**Figure 2:**
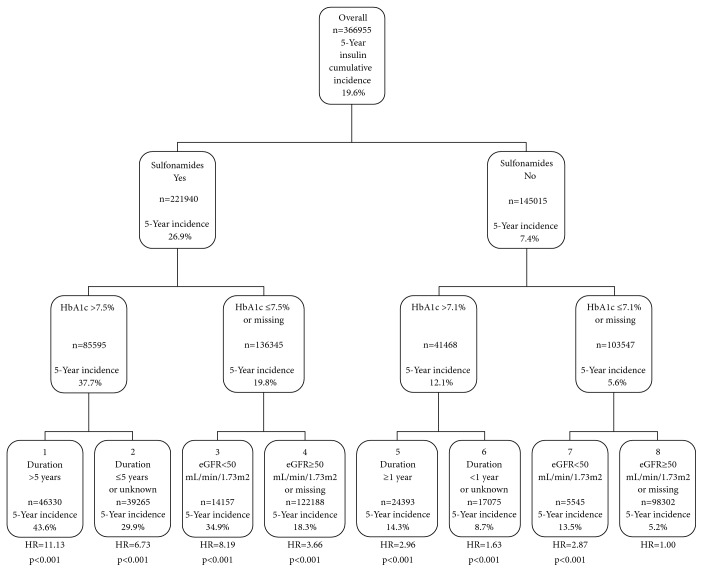
Hierarchical tree of insulin treatment initiation risk during follow-up.

Finally, by applying a tree analysis model, we identified eight patient subgroups at different risks for starting insulin treatment during follow-up; the strongest variable in terms of risk differentiation was sulfonylurea utilization. In fact, the lowest risk for starting insulin treatment was found in patients on other drugs and with HbA1c ≤ 7.1% and eGFR ≥ 50 ml/min/1.73 m^2^ at baseline. When taking them as a reference (class #8), the incidence of insulin initiation progressively increased across classes down to #1, which was mostly including patients on sulphonylurea, with HbA1c > 7.5% and disease duration > 5 years (Figure 2).

## 4. Discussion

In this report, we describe the risk profile for insulin treatment initiation in real-life conditions in a large population of patients with T2DM attending diabetes centers in Italy with an average follow-up of 42 months (21–67) and according to age and kidney function. Our data show a cumulative incidence of insulin treatment initiation on 5-year follow-up of 15%.

The ADA guidelines [6] suggest hyperglycemia to be treated as early as possible, even with insulin in specific cases, to address T2DM individualized targets. However, perceived quality of life deteriorates and major effects on costs, organization of care, and lifestyle occur after insulin enters patients' treatment regimen. For these reasons, it is important to identify early parameters predicting insulin treatment initiation.

Other previously performed real-life studies sought an answer to this question as well, but unfortunately, they involved a small series of patients, analyzed different care settings, and took only a few parameters into consideration. In the Netherlands study, the cumulative incidence of the need for an insulin switch was 36% over a 4–5-year period [14]. After nine years of follow-up in the United Kingdom Prospective Diabetes Study (UKPDS), 30% patients had switched to insulin treatment [15]. In another study, insulin therapy was started in 29.7% patients over a 2-year period [16]. In a population-based cohort study among elderly persons in Quebec, the insulin switch rate was 9.7 cases per 1000 patient-years [17], which appears very low. In the Sweden study, 25% patients with type 2 diabetes had insulin prescribed within 6 years of starting oral hypoglycemic agents and this figure rose to 42% within 10 years, corresponding to an annual rate of insulin initiation of 4% [8]. In the Australian Fremantle Diabetes Study, 15% patients had switched to insulin treatment within 5 years of follow-up [18]. The retrospective Scottish study estimated that 5.8% of those on oral hypoglycemic agents would start insulin each subsequent year within a median of 1.6 years [19].

The rate of insulin initiation described in various studies appears to be rather heterogeneous. In our opinion, this aspect can be related to the huge differences among related publications in terms of patient selection criteria, study parameters (clinical/biochemical variables, complications, and comorbidities), care settings, developing [6] or developed country daily practice habits, patient education, and quality of care of different health systems. On the contrary, in our view, it is vital to identify early predictors of insulin start: known duration of diabetes, low HDL-cholesterol, high HbA1c, BMI, triglycerides, and LDL-cholesterol values, as well as lipid-lowering treatment, eGFR, and retinopathy. A previous use of sulphonylurea, either alone or even more strongly when combined with metformin, was also found to predict the initiation of insulin therapy. The strong association of use of these drugs with the initiation of insulin treatment could be interpreted as a marker of a more complex disease and/or a loss of beta cell function with time, although a causative role cannot be excluded.

It is known that sulphonylureas act by activating insulin release from the pancreatic beta cell. However, with long-term use, there is a progressive decrease in their effectiveness. This loss of effect is in part due to a reduction in insulin-producing capacity by the pancreatic beta cell, as shown by the UKPDS study [20]. In addition, among several hypoglycemic drugs which may influence beta cell function, secretagogues appear to be the most prone to failure [4]. In fact, sulfonylurea-mediated hyperexcitation of beta cell may trigger excitotoxic reactions leading to increased rates of beta cell apoptosis [21]. As a result, beta cell mass decreases, and this is seen as the major cause of the developing insulin deficiency [22]. Consequently, control of hyperglycemia is transitory with sulphonylureas and no durable effect on HbA1c is observed.

Although experimental randomized prospective studies show that insulin therapy can be safe and efficacious in improving glycemic control in T2DM [15, 23, 24], little is known about factors associated with switching from noninsulin to insulin therapy in routine practice. Goddijn prospectively studied a cohort of patients with T2DM referred by general practitioners to an outpatient department for consideration for insulin therapy. They found that, similarly to our finding, switchers had a higher HbA1c and a lower BMI [25]. Ringborg et al. also performed a retrospective study on a population-based cohort of patients with T2DM within the Swedish RECAP-DM study for the initiation of insulin therapy. They also found that switchers had a higher HbA1c [8]. Spoelstra et al. [14] also found a higher HbA1c in patients initiating insulin therapy. Younger age and female gender were suggested by some researchers [19, 26] as factors associated with insulin initiation but could not be confirmed by others [17] including ourselves; this warrants further investigation. The high HbA1c levels at the beginning of insulin therapy may also be seen as a sign of therapeutic inertia, since all over the world, as demonstrated by the TREAT study, insulin therapy is usually started only at very high HbA1c levels [27].

Among measured biochemical factors, higher triglyceride levels were found to be associated with long-term oral hyperglycemic agent failure, and in our hands, Cox regression confirmed triglycerides to be a strong predictor for future insulin requirement. This is a major point, because hypertriglyceridemia may serve as a marker of reduced insulin production or activity and may indicate the presence of lipotoxicity, which can lead to beta cell loss [28], especially in the presence of hyperglycemia [29].

In our sample, use of statins seems to protect from insulin therapy initiation. This data is in accord with Yee et al. who found in a large cohort including 10996 T2DM patients, new users of oral antidiabetic agents, that use of statins was associated with a delay in starting insulin treatment [30]. Although the role of statins in modulating the risk of diabetes onset is still debated, Freeman et al. have reported that use of pravastatin was associated with decrease of diabetes development postulating that the anti-inflammatory actions of statins might decrease the proinflammatory milieu that predisposes to insulin resistance and diabetes [31]. It seems plausible that the same mechanisms of action would operate in patients with established diabetes and lead to a delay in the need for starting insulin treatment.

In contrast with our results, previous data suggest that eGFR decline is a significant predictor of insulin treatment start in patients treated with metformin, but not in those receiving sulphonylurea in general practice. This may depend on the limited number of patients studied by Kostev et al. [10] as well as on a different care setting. In fact, in Germany, general practitioners use metformin less extensively and recur to insulin earlier than in Italy. Culturally driven differences in therapeutic attitudes in different countries may justify the observed heterogeneity among studies' results. Thus, for example, in a recently published report from the AMD Annals initiative in Italy, a large proportion of patients with T2D were still receiving metformin despite eGFR values < 30 ml/min/1.73 m^2^[32].

Moreover, yearly incidence of insulin switch might erroneously suggest a naturally occurring, somewhat age-dependent, effect. This is not the case, as the statistics behind reflects a “time-to-event” or survival analysis where estimated absolute risk increases with the increase of the time interval. In greater detail, yearly incidence of insulin treatment initiation did not depend on the length of follow-up per se because each patient was analyzed until the end of his own time off insulin and exited the group thereafter. For example, a patient with 2-year follow-up was analyzed only up to the 2nd year and was excluded from subsequent analysis.

The tree analysis allowed us to investigate also the interaction between several clinical variables and their hierarchical impact on insulin treatment initiation and proved useful to provide physicians with clinically relevant hints. The results of this analysis clearly show that patients on sulphonylurea with high HbA1c, eGFR below 50 ml/min/1.73 m^2^, and diabetes duration longer than 5 years are at very high risk to start insulin treatment during follow-up.

We believe in the high value of identifying predictors of insulin treatment. This valuable information will allow clinicians and patients to apply preventive strategies and operate aiming to postpone an insulin therapy approach.

Our study has some limitations. First of all, data regarding the entire 5-year follow-up period (ranging from 6 to 96 months) were available for most but not all patients because this survey was realized under real-life conditions and a variable amount of missing data occurred for each patient at any steps of the follow-up. Nevertheless, we believe that this may have been counterbalanced by the very large sample size of our study. Furthermore, our data may not be applicable to the population of patients with T2DM at large, as the vast majority of participants were of white origin. Ethnicity has previously been shown to bear some impact on the risk of developing renal complications [33]. Finally, recorded therapeutic regimens largely reflect clinical practice attitudes during the time period of our study, which starts at the very beginning of incretin utilization and closes off just before SGLT2 inhibitor availability. As a consequence, only a minority of patients were taking incretins and none was on SGLT2 inhibitors.

However, we should mention the large size of the study cohort and the homogeneous geographical distribution of the recruiting centers as well as the relatively long follow-up period, which once again contribute in making the study cohort a good representation of real-life clinical practice.

## 5. Conclusions

In conclusion, even if patients with T2DM are characterized by different rates of disease progression and the clinical presentation of the disease could lead to insulin treatment initiation per se, the analysis of the collected data allows us to partly respond to typical patient's question “*will I need insulin?*,” which originated in this paper. Multivariate analysis identified several significant predictors of insulin treatment initiation: diabetes duration, HbA1c, triglyceride and low HDL-C values, the presence of retinopathy or renal function deterioration, and sulphonylurea utilization (the risk being approximately 3 times up greater in the latter case). Opposite to that, LDL-C, lipid-lowering treatment, and normal weight/overweight seemed to be protective.

Notably, the tree analysis sheds light upon the interaction between those variables and thus defined the hierarchical impact of these variables on insulin treatment initiation, identifying T2DM patients on sulphonylurea, with high HbA1c, eGFR below 50 ml/min/1.73 m^2^, and at least 5 years of disease duration as those at very high risk to start insulin treatment.

We have to stick to this real-life picture, of course, until enough data are collected from patients treated with innovative medications which are expected to improve beta cell survival and further delay treatment-related insulin requirement.

## Figures and Tables

**Table 1:** Baseline clinical characteristics of whole population and grouped by insulin treatment at follow-up.

	All	No insulin	Insulin	*p*
	*n* = 366955	*n* = 312735	*n* = 54220	
Male sex	204940 (55.8%)	175240 (56.0%)	29700 (54.8%)	<0.001
Age (years)	65 ± 11	65 ± 11	66 ± 11	<0.001
Duration of diabetes (years)	7 ± 8	6 ± 7	10 ± 9	<0.001
BMI (kg/m^2^)	30 ± 5	30 ± 5	29 ± 5	<0.001
Waist circumference (cm)	103 ± 13	103 ± 12	103 ± 13	0.205
Serum creatinine (mg/dl)	0.95 ± 0.53	0.95 ± 0.52	1.00 ± 0.60	<0.001
eGFR (ml/min/1.73 m^2^)	78 ± 19	79 ± 19	75 ± 21	<0.001
eGFR < 60 ml/min/1.73 m^2^	63635 (17.3%)	50620 (16.2%)	13015 (24.0%)	<0.001
Albuminuria	31814 (21.1%)	25661 (20.1%)	6153 (27.4%)	<0.001
Microalbuminuria	27125 (18.0%)	22125 (17.3%)	5000 (22.2%)	<0.001
Macroalbuminuria	4689 (3.1%)	3536 (2.8%)	1153 (5.1%)	<0.001
Serum uric acid (mg/dl)	5.5 ± 1.9	5.5 ± 1.9	5.4 ± 1.8	<0.001
Serum uric acid in the quintile	31171 (18.9%)	26503 (18.8%)	4668 (19.0%)	<0.001
HbA1c (%)	7.3 ± 1.6	7.2 ± 1.5	8.0 ± 1.6	<0.001
HbA1c ≥ 7%	179176 (50.2%)	140643 (46.2%)	38533 (72.9%)	<0.001
Total cholesterol (mg/dl)	198 ± 43	198 ± 42	198 ± 43	<0.001
Triglycerides (mg/dl)	156 ± 121	154 ± 118	166 ± 139	<0.001
Triglycerides ≥ 150 mg/dl	126931 (38.7%)	106597 (38.0%)	20334 (42.9%)	<0.001
HDL (mg/dl)	50 ± 14	50 ± 14	50 ± 14	<0.001
HDL < 40 mg/dl (M), <50 mg/dl (F)	108975 (34.0%)	92748 (33.8%)	16227 (35.0%)	<0.001
LDL (mg/dl)	118 ± 36	119 ± 36	117 ± 36	<0.001
LDL ≥ 100 mg/dl	216751 (68.6%)	186353 (68.9%)	30398 (67.0%)	<0.001
Systolic BP (mmHg)	140 ± 19	140 ± 19	142 ± 20	<0.001
Diastolic BP (mmHg)	81 ± 10	81 ± 10	81 ± 10	<0.001
BP ≥ 140/85 mmHg	192966 (62.4%)	163268 (61.9%)	29698 (64.9%)	<0.001
Nonproliferative retinopathy	19410 (5.3%)	14516 (4.6%)	4894 (9.0%)	<0.001
Proliferative retinopathy	4479 (1.2%)	3183 (1.0%)	1296 (2.4%)	<0.001
Smokers	33276 (17.2%)	28479 (17.1%)	4797 (18.2%)	<0.001
Lipid-lowering treatment	140692 (38.3%)	121976 (39.0%)	18716 (34.5%)	<0.001
Treatment with statins	127489 (34.7%)	110893 (35.5%)	16596 (30.6%)	<0.001
Treatment with fibrates	8399 (2.3%)	6985 (2.2%)	1414 (2.6%)	<0.001
Antihypertensive treatment	214395 (58.4%)	182658 (58.4%)	31737 (58.5%)	0.003
Treatment with ACE-Is/ARBs	177232 (48.3%)	150950 (48.3%)	26282 (48.5%)	0.003
Aspirin	60816 (16.6%)	51548 (16.5%)	9268 (17.1%)	<0.001
Diet	42056 (11.5%)	40220 (12.9%)	1836 (3.4%)	<0.001
Biguanides and sulphonylureas	159664 (43.5%)	124017 (39.7%)	35647 (65.7%)	—
Biguanides	99718 (27.2%)	93745 (30.0%)	5973 (11.0%)	<0.001
Sulphonylureas	62276 (17.0%)	51751 (16.5%)	10525 (19.4%)	<0.001
Glitazones	3241 (0.9%)	3002 (1.0%)	239 (0.4%)	<0.001

Mean ± SD or absolute frequency (percentage). ACE-Is: angiotensin-converting enzyme inhibitors; ARBs: angiotensin II receptor antagonists; ALT: alanine transaminase; AST: aspartate aminotransferase; BMI: body mass index; BP: blood pressure; eGFR: estimated glomerular filtration rate; GGT: gamma-glutamyltransferase; HbA1c: glycated haemoglobin; HDL: high-density lipoprotein cholesterol; LDL: low-density lipoprotein cholesterol. Serum uric acid gender specific quintile: females > 6.3 mg/dl and males > 6.8 mg/dl. Patients' baseline missing data: duration of diabetes 34769 (9.5%), BMI 42789 (11.7%), waist circumference 281370 (76.7%), albuminuria 216481 (59.0%), serum uric acid 201675 (55.0%), HbA1c 9865 (2.7%), total cholesterol 35355 (9.6%), triglycerides 38722 (10.6%), HDL 46234 (12.6%), LDL 51112 (13.9%), systolic and diastolic BP (mmHg) 57478 (15.7%), and smoking status 173783 (47.4%).

**Table 2:** Multivariate Cox regression analyses for insulin treatment at follow-up.

Patients with complete data	Model 1		Model 2		Model 3		Model 4	
	*N* = 323769		*N* = 236412		*N* = 73069		*N* = 44611	
	Hazard ratio	*p*	Hazard ratio	*p*	Hazard ratio	*p*	Hazard ratio	*p*
Male sex	1.037	<0.001	1.035	0.002	0.993	0.724	0.985	0.587
Age (by 10 years)	0.955	<0.001	0.958	<0.001	0.970	0.014	0.970	0.052
Duration of diabetes (by 10 years)	1.315	<0.001	1.332	<0.001	1.350	<0.001	1.363	<0.001
HbA1c (by 1%)	1.264	<0.001	1.260	<0.001	1.243	<0.001	1.241	<0.001
Nonproliferative retinopathy	1.287	<0.001	1.288	<0.001	1.256	<0.001	1.277	<0.001
Proliferative retinopathy	1.436	<0.001	1.392	<0.001	1.441	<0.001	1.547	<0.001
eGFR below 90 (by 10 ml/min/1.73 m^2^)	1.157	<0.001	1.139	<0.001	1.122	<0.001	1.121	<0.001
Triglycerides ≥ 150 mg/dl			1.079	<0.001	1.060	0.007	1.095	0.001
HDL < 40 mg/dl (M), <50 mg/dl (F)			1.100	<0.001	1.103	<0.001	1.124	<0.001
LDL ≥ 100 mg/dl			0.819	<0.001	0.863	<0.001	0.861	<0.001
Systolic/diastolic BP ≥ 140/85 mmHg			0.950	<0.001	0.945	0.009	0.949	0.059
*Albuminuria*								
Normoalbuminuria					*Reference*		*Reference*	
Microalbuminuria					1.366	<0.001	1.414	<0.001
Macroalbuminuria					2.028	<0.001	2.210	<0.001
*BMI*								
<27 kg/m^2^							*Reference*	
27–30 kg/m^2^							0.882	<0.001
>30 kg/m^2^							0.834	<0.001
Serum uric acid in the top quintile							1.046	0.209
Lipid-lowering treatment	0.912	<0.001	0.901	<0.001	0.867	<0.001	0.855	<0.001
Antihypertensive treatment	1.002	0.819	0.999	0.916	1.000	0.998	1.020	0.501
Aspirin	1.007	0.598	1.010	0.515	1.002	0.948	1.002	0.966
*Antidiabetic treatment*								
Diet	*Reference*		*Reference*		*Reference*		*Reference*	
Biguanides and sulphonylureas	3.860	<0.001	3.963	<0.001	3.581	<0.001	3.511	<0.001
Sulphonylureas	2.874	<0.001	2.950	<0.001	2.806	<0.001	2.804	<0.001
Glitazones	1.649	<0.001	1.691	<0.001	1.469	0.011	1.534	0.031
Biguanides	1.286	<0.001	1.283	<0.001	1.188	0.004	1.209	0.012

Hazard ratios from multivariate Cox proportional hazard models for insulin treatment during follow-up.

## Data Availability

The data are freely available; the data used to support the findings of this study are included within the article and within the supplementary information files.

## Supplementary Materials

Supplementary 1 Supplementary Figure 1: flow diagram for the selection of study patients.

Supplementary 2 Supplementary Figure 2: cumulative incidence of insulin treatment initiation during follow-up, as related to glucose-lowering treatment. Sulfonamides = sulphonylurea; biguanides = metformin.

Supplementary 3 Supplementary Figure 3: incidence of insulin treatment initiation in relation to eGFR range.

Supplementary 4 Supplementary Table 1: this table shows baseline clinical characteristics of patients studied by the year of insulin treatment after baseline.

Supplementary 5 Supplementary Table 2: this table shows baseline clinical characteristics of patients studied by antidiabetic treatment.
