# Supplementary material for: Five-Year Predictors of Insulin Initiation in People with Type 2 Diabetes under Real-Life Conditions
Source: J Diabetes Res. 2018 Sep 19;2018:7153087. doi: 10.1155/2018/7153087 (PMC6169213; doi:10.1155/2018/7153087)
Supplement: Supplementary 1 — Supplementary Figure 1: flow diagram for the selection of study patients. [file 7153087.f1.ppt]

## Slide 1
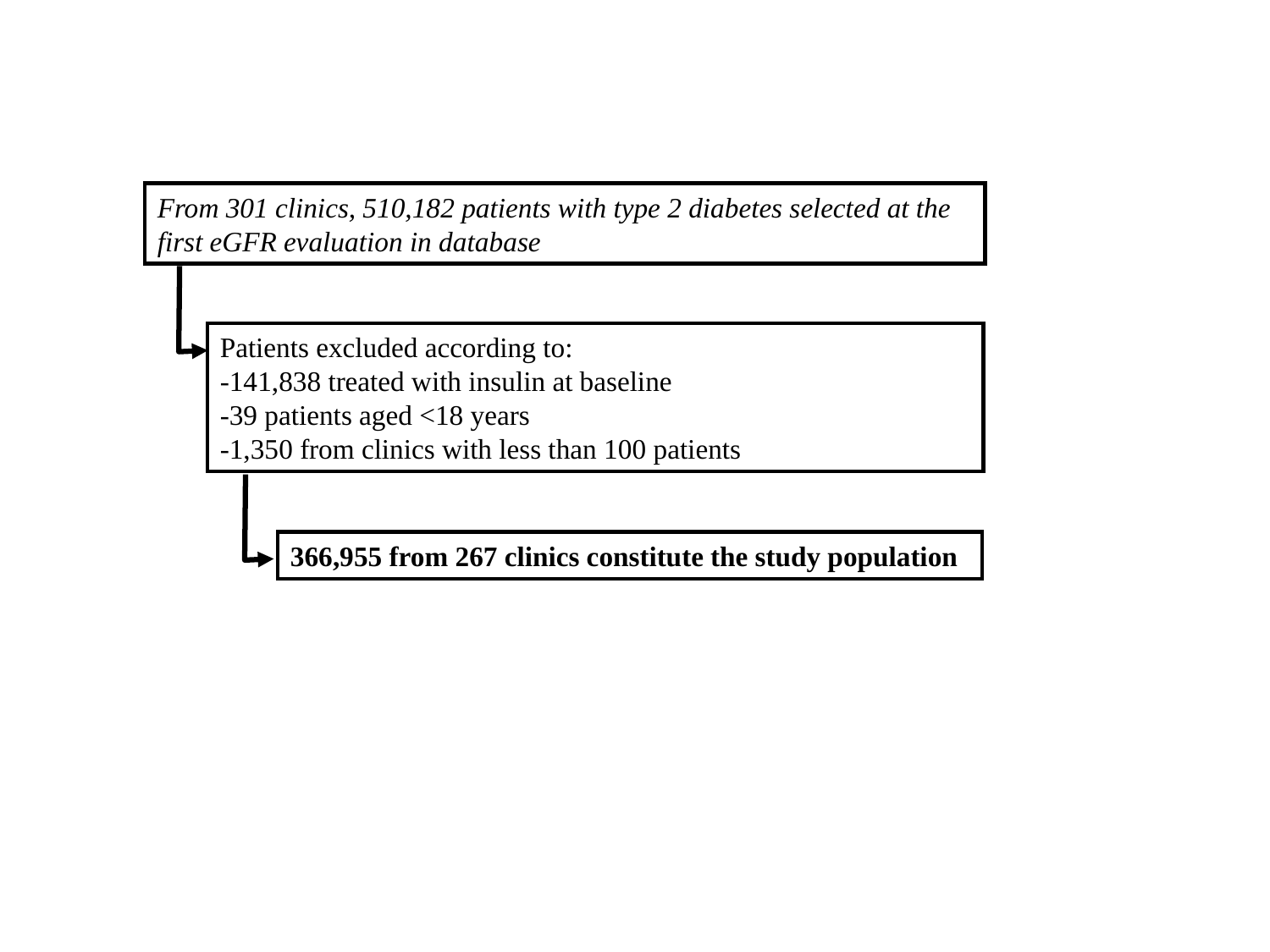

From 301 clinics, 510,182 patients with type 2 diabetes selected at the first eGFR evaluation in database
Patients excluded according to:
-141,838 treated with insulin at baseline
-39 patients aged <18 years
-1,350 from clinics with less than 100 patients
366,955 from 267 clinics constitute the study population
