# Supplementary figures and images for: Five-Year Predictors of Insulin Initiation in People with Type 2 Diabetes under Real-Life Conditions
Source: J Diabetes Res. 2018 Sep 19;2018:7153087. doi: 10.1155/2018/7153087 (PMC6169213; doi:10.1155/2018/7153087)

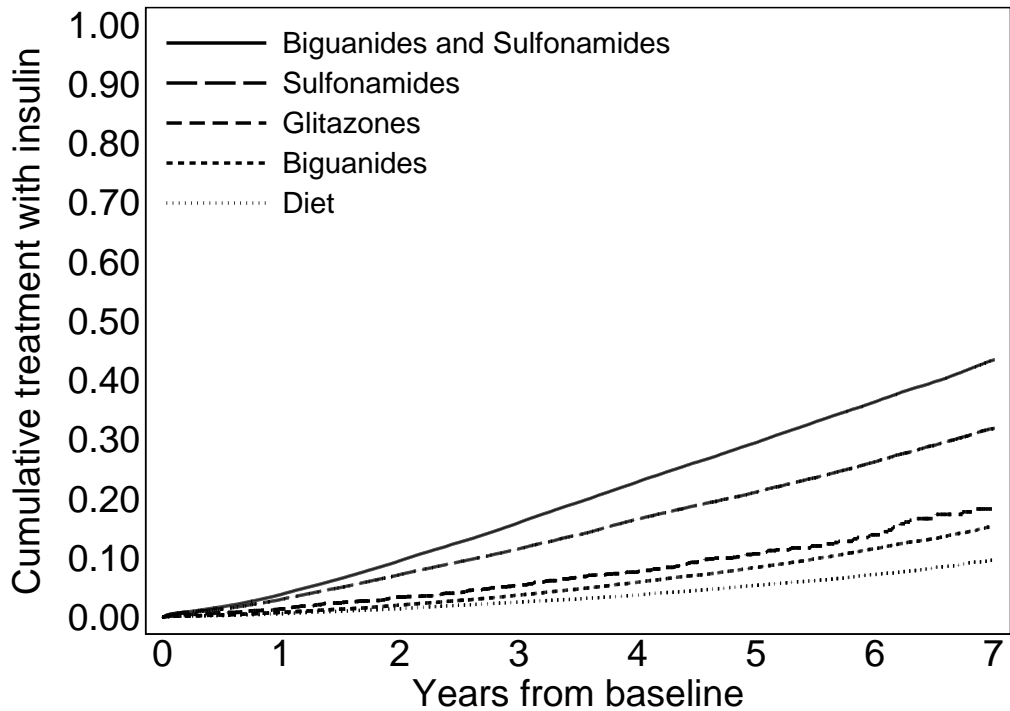

Supplement: Supplementary 2 — Supplementary Figure 2: cumulative incidence of insulin treatment initiation during follow-up, as related to glucose-lowering treatment. Sulfonamides = sulphonylurea; biguanides = metformin. [file 7153087.f2.pdf]

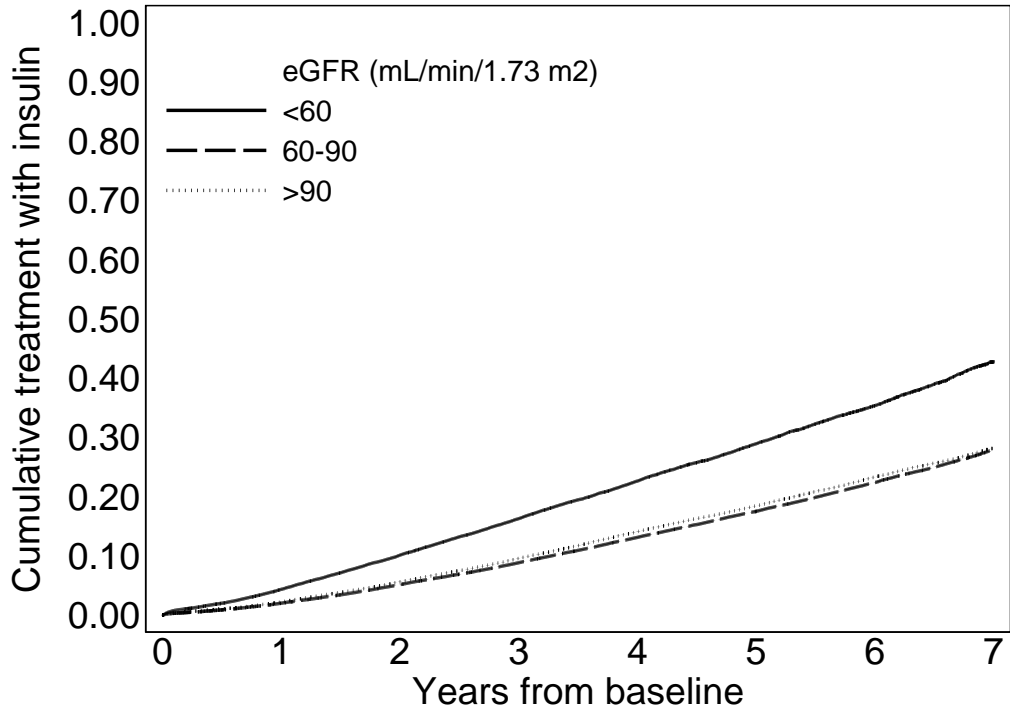

Supplement: Supplementary 3 — Supplementary Figure 3: incidence of insulin treatment initiation in relation to eGFR range. [file 7153087.f3.pdf]
