# Supplementary material for: Five-Year Predictors of Insulin Initiation in People with Type 2 Diabetes under Real-Life Conditions
Source: J Diabetes Res. 2018 Sep 19;2018:7153087. doi: 10.1155/2018/7153087 (PMC6169213; doi:10.1155/2018/7153087)
Supplement: Supplementary 4 — Supplementary Table 1: this table shows baseline clinical characteristics of patients studied by the year of insulin treatment after baseline. [file 7153087.f4.docx]

**Supplementary Table 1 Baseline clinical characteristics by the year of insulin treatment after baseline.**

|  |  | **Years from baseline to insulin treatment** | | | | | **No insulin** |
| --- | --- | --- | --- | --- | --- | --- | --- |
|  | **All** | **<1** | **1 to 2** | **2 to 3** | **3 to 4** | **4 to 5** | **at 5^th^ year** |
|  | **n=177583** | **n=8664** | **n=10622** | **n=9858** | **n=8801** | **n=6604** | **n=133034** |
| Male sex | 99354 (55.9%) | 4664 (53.8%) | 5796 (54.6%) | 5474 (55.5%) | 4856 (55.2%) | 3606 (54.6%) | 74958 (56.3%) |
| Age (years) | 65±10 | 68±12 | 67±11 | 66±11 | 66±11 | 66±11 | 64±10 |
| Duration of diabetes (years) | 8±8 | 11±9 | 11±9 | 10±8 | 10±8 | 10±8 | 7±7 |
| BMI (Kg/m^2^) | 30±5 | 29±5 | 29±5 | 29±5 | 30±5 | 30±5 | 30±5 |
| Waist circumference (cm) | 103±12 | 102±13 | 103±14 | 103±13 | 103±13 | 102±13 | 103±12 |
| Serum creatinine (mg/dL) | 0.96±0.52 | 1.07±0.74 | 1.02±0.62 | 1.01±0.65 | 0.98±0.57 | 0.97±0.49 | 0.94±0.47 |
| eGFR (mL/min/1.73 m^2^) | 78±19 | 72±24 | 74±22 | 75±22 | 76±20 | 77±20 | 79±18 |
| eGFR<60 mL/min/1.73 m^2^ | 30199 (17.0%) | 2622 (30.3%) | 2848 (26.8%) | 2419 (24.5%) | 1939 (22.0%) | 1394 (21.1%) | 18977 (14.3%) |
| Albuminuria | 15551 (20.7%) | 1236 (33.0%) | 1298 (30.4%) | 1096 (27.6%) | 898 (25.4%) | 709 (25.6%) | 10314 (18.1%) |
| Microalbuminuria | 13355 (17.7%) | 959 (25.6%) | 1050 (24.6%) | 881 (22.2%) | 733 (20.8%) | 599 (21.6%) | 9133 (16.0%) |
| Macroalbuminuria | 2196 (2.9%) | 277 (7.4%) | 248 (5.8%) | 215 (5.4%) | 165 (4.7%) | 110 (4.0%) | 1181 (2.1%) |
| Serum uric acid (mg/dL) | 5.4±1.8 | 5.4±2.0 | 5.4±1.9 | 5.4±1.8 | 5.4±1.8 | 5.4±1.8 | 5.4±1.8 |
| Serum uric acid in the top quintile | 13956 (18.0%) | 865 (20.6%) | 916 (19.0%) | 855 (19.0%) | 691 (18.3%) | 591 (19.8%) | 10038 (17.6%) |
| HbA1c (%) | 7.4±1.6 | 8.5±1.7 | 8.1±1.6 | 8.0±1.6 | 7.9±1.5 | 7.7±1.5 | 7.1±1.5 |
| HbA1c≥7% | 93076 (53.8%) | 6982 (82.1%) | 7900 (76.3%) | 7015 (72.9%) | 6081 (71.1%) | 4378 (68.0%) | 60720 (46.9%) |
| Total cholesterol (mg/dL) | 200±42 | 193±46 | 196±44 | 196±44 | 199±42 | 200±41 | 202±41 |
| Triglycerides (mg/dL) | 156±123 | 172±168 | 169±135 | 168±139 | 167±149 | 161±121 | 152±116 |
| Triglycerides ≥150 mg/dl | 60829 (38.8%) | 3436 (45.0%) | 4109 (44.3%) | 3778 (43.7%) | 3256 (42.4%) | 2365 (40.8%) | 43885 (37.3%) |
| HDL (mg/dL) | 51±14 | 49±15 | 49±14 | 50±14 | 50±14 | 51±14 | 51±14 |
| HDL <40M <50F mg/dL | 48499 (31.7%) | 2904 (38.9%) | 3405 (37.4%) | 2970 (35.1%) | 2553 (34.1%) | 1886 (33.1%) | 34781 (30.3%) |
| LDL (mg/dL) | 120±36 | 112±38 | 114±37 | 115±36 | 118±37 | 119±35 | 122±35 |
| LDL ≥100 mg/dL | 107634 (71.5%) | 4485 (61.2%) | 5675 (63.6%) | 5410 (65.4%) | 5051 (69.0%) | 3974 (71.4%) | 83039 (73.4%) |
| Systolic BP (mmHg) | 141±19 | 141±20 | 142±20 | 142±20 | 142±20 | 142±20 | 141±19 |
| Diastolic BP (mmHg) | 81±10 | 80±10 | 81±10 | 81±10 | 82±10 | 82±10 | 82±10 |
| BP≥140/85 mmHg | 96292 (64.8%) | 4538 (61.6%) | 5745 (64.1%) | 5359 (64.7%) | 4886 (65.9%) | 3709 (66.6%) | 72055 (65.0%) |
| Non-proliferative retinopathy | 10995 (6.2%) | 971 (11.2%) | 1089 (10.3%) | 949 (9.6%) | 710 (8.1%) | 497 (7.5%) | 6779 (5.1%) |
| Proliferative retinopathy | 2555 (1.4%) | 282 (3.3%) | 307 (2.9%) | 219 (2.2%) | 176 (2.0%) | 125 (1.9%) | 1446 (1.1%) |
| Smokers | 15064 (17.0%) | 748 (18.9%) | 992 (18.9%) | 915 (18.6%) | 815 (18.4%) | 595 (17.9%) | 10999 (16.5%) |
| Lipid-lowering treatment | 63644 (35.8%) | 2927 (33.8%) | 3861 (36.3%) | 3377 (34.3%) | 3099 (35.2%) | 2261 (34.2%) | 48119 (36.2%) |
| Treatment with statins | 57327 (32.3%) | 2602 (30.0%) | 3408 (32.1%) | 3008 (30.5%) | 2759 (31.3%) | 1989 (30.1%) | 43561 (32.7%) |
| Treatment with fibrates | 3988 (2.2%) | 205 (2.4%) | 310 (2.9%) | 259 (2.6%) | 238 (2.7%) | 184 (2.8%) | 2792 (2.1%) |
| Antihypertensive treatment | 101708 (57.3%) | 4916 (56.7%) | 6364 (59.9%) | 5732 (58.1%) | 5215 (59.3%) | 3954 (59.9%) | 75527 (56.8%) |
| Treatment with ACE-Is/ARBs | 84056 (47.3%) | 4057 (46.8%) | 5268 (49.6%) | 4750 (48.2%) | 4278 (48.6%) | 3294 (49.9%) | 62409 (46.9%) |
| Aspirin | 29205 (16.4%) | 1163 (13.4%) | 1906 (17.9%) | 1780 (18.1%) | 1595 (18.1%) | 1150 (17.4%) | 21611 (16.2%) |
| Diet | 20102 (11.3%) | 242 (2.8%) | 283 (2.7%) | 313 (3.2%) | 264 (3.0%) | 279 (4.2%) | 18721 (14.1%) |
| Biguanides and sulphonylureas | 82766 (46.6%) | 5817 (67.1%) | 7226 (68.0%) | 6672 (67.7%) | 5805 (66.0%) | 4262 (64.5%) | 52984 (39.8%) |
| Biguanides | 40650 (22.9%) | 767 (8.9%) | 933 (8.8%) | 1001 (10.2%) | 981 (11.1%) | 835 (12.6%) | 36133 (27.2%) |
| Sulphonylureas | 32724 (18.4%) | 1794 (20.7%) | 2128 (20.0%) | 1832 (18.6%) | 1715 (19.5%) | 1197 (18.1%) | 24058 (18.1%) |
| Glitazones | 1341 (0.8%) | 44 (0.5%) | 52 (0.5%) | 40 (0.4%) | 36 (0.4%) | 31 (0.5%) | 1138 (0.9%) |

Mean±SD or absolute frequency (percentage). Data by the year from baseline to insulin treatment before the 5^th^ in comparison to patients free from treatment. Patients with follow-up shorter than 5 years not treated by the end of follow-up were excluded. Patients' baseline missing data: duration of diabetes 14197 (8%), BMI 22230 (12.5%), Waist circumference 149646 (84.3%), Albuminuria 102302 (57.6%), Serum uric acid 100148 (56.4%), HbA1c 4587 (2.6%), Total cholesterol 19144 (10.8%), Triglycerides 20964 (11.8%), HDL 24572 (13.8%), LDL 7051 (15.2%), Systolic and diastolic BP (mmHg) 29097 (16.4%), and smoking status 88946 (50.1%).
