# Supplementary material for: Five-Year Predictors of Insulin Initiation in People with Type 2 Diabetes under Real-Life Conditions
Source: J Diabetes Res. 2018 Sep 19;2018:7153087. doi: 10.1155/2018/7153087 (PMC6169213; doi:10.1155/2018/7153087)
Supplement: Supplementary 5 — Supplementary Table 2: this table shows baseline clinical characteristics of patients studied by antidiabetic treatment. [file 7153087.f5.docx]

**Supplementary Table 2 Baseline clinical characteristics by antidiabetic treatment~~.~~**

|  | **Biguanides and Sulphonamides** | **Sulphonamides** | **Biguanides** | **Glitazones** | **Diet** |
| --- | --- | --- | --- | --- | --- |
|  | **n=159664** | **n=62276** | **n=99718** | **n=3241** | **n=42056** |
| Male sex | 87460 (54.8%) | 34813 (55.9%) | 55972 (56.1%) | 1806 (55.7%) | 24889 (59.2%) |
| Age (years) | 66±10 | 70±11 | 62±10 | 61±10 | 65±11 |
| Duration of diabetes (years) | 9±8 | 8±8 | 4±6 | 5±6 | 4±5 |
| BMI (Kg/m^2^) | 30±5 | 28±5 | 31±5 | 31±6 | 29±5 |
| Waist circumference (cm) | 104±12 | 99±12 | 105±12 | 105±13 | 101±12 |
| Serum creatinine (mg/dL) | 0.94±0.53 | 1.06±0.65 | 0.91±0.45 | 0.93±0.43 | 0.97±0.54 |
| eGFR (mL/min/1.73 m^2^) | 79±18 | 71±21 | 82±18 | 82±19 | 78±19 |
| eGFR<60 mL/min/1.73 m^2^ | 26465 (16.6%) | 18983 (30.5%) | 10965 (11.0%) | 446 (13.8%) | 6776 (16.1%) |
| Albuminuria | 15876 (23.7%) | 5153 (22.1%) | 8010 (19.4%) | 255 (19.9%) | 2520 (14.4%) |
| Microalbuminuria | 13467 (20.1%) | 4242 (18.2%) | 7023 (17.0%) | 230 (18.0%) | 2163 (12.4%) |
| Macroalbuminuria | 2409 (3.6%) | 911 (3.9%) | 987 (2.4%) | 25 (2.0%) | 357 (2.0%) |
| Serum uric acid (mg/dL) | 5.3±1.9 | 5.5±2.0 | 5.5±1.9 | 5.5±2.2 | 5.6±1.7 |
| Serum uric acid in the top quintile | 12017 (16.9%) | 5605 (21.4%) | 9250 (19.7%) | 259 (18.3%) | 4040 (20.5%) |
| HbA1c (%) | 7.7±1.6 | 7.1±1.5 | 7.1±1.5 | 7.2±1.5 | 6.4±1.0 |
| HbA1c≥7% | 99591 (64.0%) | 27783 (46.1%) | 42554 (44.0%) | 1470 (46.8%) | 7778 (18.8%) |
| Total cholesterol (mg/dL) | 196±42 | 197±42 | 199±43 | 204±44 | 204±41 |
| Triglycerides (mg/dL) | 160±124 | 144±103 | 161±132 | 174±135 | 141±101 |
| Triglycerides ≥150 mg/dl | 58296 (40.8%) | 18218 (34.0%) | 37204 (40.7%) | 1351 (45.4%) | 11862 (31.9%) |
| HDL (mg/dL) | 50±14 | 51±15 | 49±14 | 49±14 | 52±14 |
| HDL <40M <50F mg/dL | 48778 (35.0%) | 16879 (32.2%) | 31831 (35.6%) | 1054 (36.4%) | 10433 (28.4%) |
| LDL (mg/dL) | 116±36 | 119±36 | 119±36 | 123±38 | 125±36 |
| LDL ≥100 mg/dL | 90618 (66.0%) | 35416 (69.1%) | 60959 (69.4%) | 2080 (72.4%) | 27678 (75.7%) |
| Systolic BP (mmHg) | 142±19 | 140±20 | 139±19 | 138±18 | 138±19 |
| Diastolic BP (mmHg) | 81±10 | 80±10 | 82±10 | 81±10 | 81±10 |
| BP≥140/85 mmHg | 87297 (64.7%) | 31649 (61.4%) | 51822 (61.1%) | 1571 (58.6%) | 20627 (58.0%) |
| Non-proliferative retinopathy | 12255 (7.7%) | 2889 (4.6%) | 3428 (3.4%) | 122 (3.8%) | 716 (1.7%) |
| Proliferative retinopathy | 3165 (2.0%) | 639 (1.0%) | 573 (0.6%) | 21 (0.6%) | 81 (0.2%) |
| Smokers | 13971 (17.5%) | 4283 (15.0%) | 10806 (18.6%) | 357 (19.5%) | 3859 (15.5%) |
| Lipid-lowering treatment | 62433 (39.1%) | 21513 (34.5%) | 40465 (40.6%) | 1401 (43.2%) | 14880 (35.4%) |
| Treatment with statins | 56417 (35.3%) | 19570 (31.4%) | 36506 (36.6%) | 1221 (37.7%) | 13775 (32.8%) |
| Treatment with fibrates | 4041 (2.5%) | 1116 (1.8%) | 2539 (2.5%) | 113 (3.5%) | 590 (1.4%) |
| Antihypertensive treatment | 93971 (58.9%) | 36258 (58.2%) | 58483 (58.6%) | 1769 (54.6%) | 23914 (56.9%) |
| Treatment with ACE-Is/ARBs | 79272 (49.6%) | 28717 (46.1%) | 49007 (49.1%) | 1491 (46.0%) | 18745 (44.6%) |
| Aspirin | 28839 (18.1%) | 10798 (17.3%) | 15743 (15.8%) | 464 (14.3%) | 4972 (11.8%) |

Mean±SD or absolute frequency (percentage).
